# Guiding principles for technical infrastructure to support computable biomedical knowledge

**DOI:** 10.1002/lrh2.10352

**Published:** 2022-11-01

**Authors:** Jamie McCusker, Leslie D. McIntosh, Chris Shaffer, Peter Boisvert, James Ryan, Vivek Navale, Umit Topaloglu, Rachel L. Richesson

**Affiliations:** ^1^ Rensselaer Polytechnic Institute Computer Science Troy New York USA; ^2^ Ripeta LLC St Louis Missouri USA; ^3^ Research Data Alliance New Jersey USA; ^4^ University of California San Francisco, Library San Francisco California USA; ^5^ Department of Learning Health Sciences University of Michigan Ann Arbor Michigan USA; ^6^ Ryan Family Practice Ludington Michigan USA; ^7^ National Institutes of Health Center for Information Technology Bethesda Maryland USA; ^8^ Wake Forest School of Medicine Cancer Biology Winston‐Salem North Carolina USA

**Keywords:** computable biomedical knowledge, FAIR, open systems

## Abstract

Over the past 4 years, the authors have participated as members of the Mobilizing Computable Biomedical Knowledge Technical Infrastructure working group and focused on conceptualizing the infrastructure required to use computable biomedical knowledge. Here, we summarize our thoughts and lay the foundation for future work in the development of CBK infrastructure, including: explaining the difference between computable knowledge and data, and contextualizing the conversation with the Learning Health Systems and the FAIR principles. Specifically, we provide three guiding principles to advance the development of CBK infrastructure: (a) Promote interoperable systems for data and knowledge to be findable, accessible, interoperable, and reusable. (b) Enable stable, trustworthy knowledge representations that are human and machine readable. (c) Computable knowledge resources should, when possible, be open. Standards supporting computable knowledge infrastructures must be open.

## 1 INTRODUCTION

Computable formats for biomedical knowledge are needed to support the continuous incorporation of evidence and emerging knowledge structures into the Learning Health System (LHS).^1^ The growth of computable biomedical knowledge (CBK) and related opportunities is driving renewed interest in knowledge representation to support CBK and the infrastructure required to disseminate and apply it to different settings. However, there are many unanswered questions and opportunities for exploration.

To fully explore the requirements for a CBK infrastructure we need to understand the lifecycle of CBK, specifically how it is created, maintained, evaluated, and integrated into the broader technical landscape. This CBK technical infrastructure will need to support both management of CBK in its various forms and the movement of CBK into practice, integration with existing systems, and appropriate and effective use.

**FIGURE 1:**
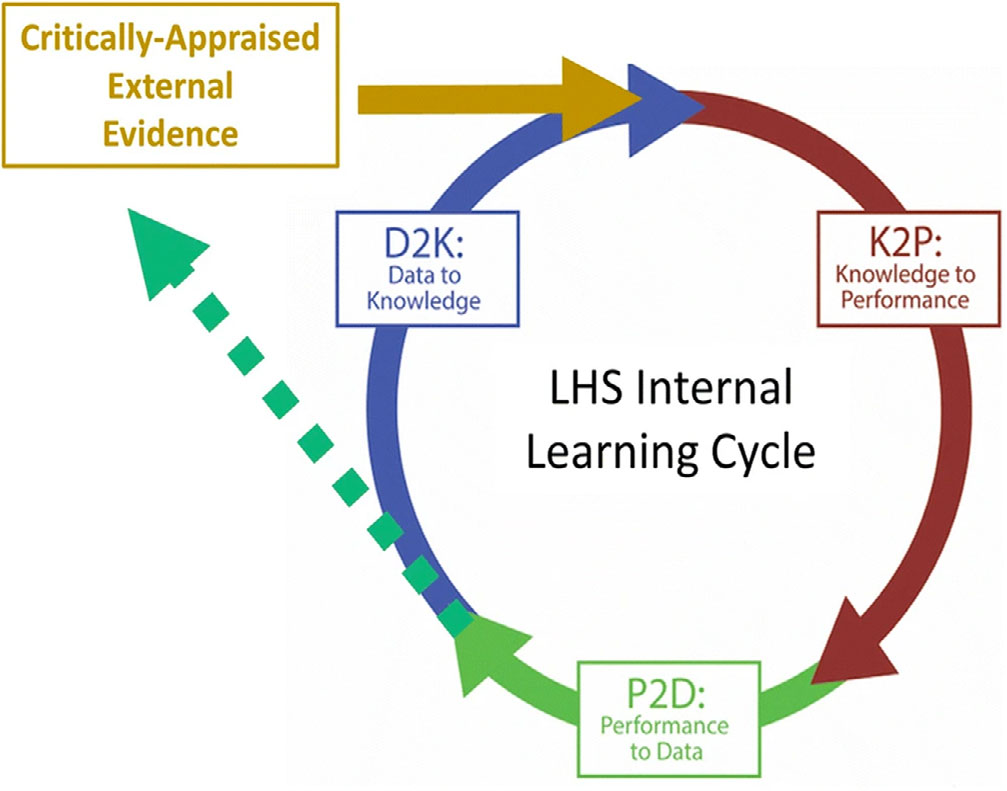
The learning cycle represents a dynamic coupling between data, knowledge, performance and learning community, to illustrate a continuous feedback loop that requires essential technical infrastructure support. Previously published in Guise et al.^1^

For 5 years, a number of interested experts from various biomedical domains have been meeting to articulate the field of CBK application, specify technical infrastructure requirements for applying CBK at scale, and develop an ecosystem for the safe and efficient application of CBK in health contexts. The purpose of this paper is to present the outcomes of these discussions and articulate the principles necessary to support this critical dimension of the learning cycle (Figure 1) infrastructure. Our group identified the principles needed for knowledge to be findable, accessible, interoperable, and reusable (FAIR),^2^ with a focus on interoperability, and generated a number of scenarios to illustrate the importance and relevance of these principles. We extend the earlier work to the domain of computable knowledge representation to bridge the gap between the principles and knowledge implementation pathways by identifying infrastructure characteristics that can lead to the dissemination of trustworthy, open knowledge objects within an LHS. We call this approach FAIR + TO.

*The MCBK Technical Standards and Infrastructure Working Group* is a self‐selected, volunteer interest group led by two co‐chairs (originally as Technical Infrastructure under LM and CS, now JM and Bruce Bray*) for the past 5 years and is supported by the University of Michigan's MCBK team. The community is open and has a listserv. The MCBK SI group has met in person three times at public meetings hosted by NLM in the summers of 2018,^3^ 2019,^4^ 2020,^5^ and has had multiple phone meetings in between. In the summer 2020 meeting, the SI workgroup continued work on this article. An average of 15 participants joined these discussions. Here, we present the culmination of these 3 years of discussions and identify current and future requirements to stimulate the development of CBK and its developing ecosystem.

## 2 CBK TECHNICAL INFRASTRUCTURE—COMPONENTS AND KEY TERMS

“Computable Biomedical Knowledge (CBK) is the result of an analytic or deliberative process about or affecting human health, that is explicit, and therefore can be represented and reasoned upon using logic, formal standards, and mathematical approaches”—MCBK Manifesto.^4^

CBK is related to, but distinctly different from, biomedical data. While CBK has a direct connection with data, managing and sharing it has its own unique needs. We see the following difference between data and knowledge. Data are the representations of information at a granular level while knowledge is data combined with processed information—such as attributes and relationships—to apply meaning to the data.^1^ Here, a database is the storage of raw elements that have no meaning without linked context, such as metadata. However, metadata alone do not provide knowledge to data, as metadata still lack meaning and need interpretation. A knowledge base, therefore, will house human and machine‐readable data objects, attributes, linkages, and interpretations that represent usable knowledge.

For computable knowledge to be used at scale and openly, infrastructure is needed. We use the definition of infrastructure used by the Invest in Open Infrastructure Initiative.^6^ CBK has a lifecycle and can be created, modified, and updated. To support this knowledge, infrastructure must have a flexible, scalable, and maintainable pipeline for continuous input, output, growth, and use of knowledge that serves as the foundation for the LHS.^1^ The importance of coupling human intelligence (eg, actual clinical data from the population) improves the accuracy of knowledge objects (Figure 1). Because knowledge evolves with new data, infrastructure should be robust and agile to accommodate the changes in scale, enhance precision in analysis, and provide services to a wide range of community needs.

The developed infrastructure should support interoperability, however, the requirements for interoperability data and knowledge are somewhat different. Interoperability in data means things like aligning format, structural metadata, and semantics, such as defined by the FAIR principles.^2^ Data itself is passive. Only supporting infrastructure can make data discoverable, linkable, transformable, shippable, archivable, and so on. While those services also apply to computable knowledge, knowledge additionally needs to be implemented, tested, integrated with external systems, updated, versioned, reused, and trusted. A knowledge infrastructure must therefore support what it does, not just what it is—as in the case with data object infrastructure.

## 3 CBK IN A GLOBAL PANDEMIC

The recent global COVID‐19 pandemic illustrates the need for CBK infrastructure and underscores the importance of multiple perspectives on how knowledge bases can be developed and applied. In the context of the COVID‐19 pandemic, the algorithms associated with the various epidemiological models^7^ are examples of CBK. During the early stages of the pandemic, data was less available and uncertainties in model predictions were significant. As the pandemic progressed more data became available, and with the inclusion of additional model parameters the confidence interval for the mortality model predictions improved.^8^ The continuous iterative LHS life cycle process shown in Figure 1 enabled the refinement of knowledge bases that resulted in the implementation of evidence‐based health care policy measures for various COVID‐19 impacted localities. During the pandemic, the magnitude and scope of CBKs produced were enormous, from modeling to clinical and translational research and the development of therapeutics. Such a gigantic enterprise resulted in the development and application of a wide range of platforms, services, and knowledge repositories. Fundamentally, an important aspect of the technical infrastructure is to enable knowledge to be FAIR. In the following paragraphs, we elaborate on the principles that can serve as the basis for CBK technical infrastructure.

## 4 PRINCIPLES OF A CBK TECHNICAL INFRASTRUCTURE

We posit that CBK infrastructure should follow the FAIR principles plus two more: Trustworthiness and Openness. We call this FAIR + TO. The MCBK Standards Working Group published a good overview of the sort of metadata needed to index CBK for FAIR + T,^9^ which we see as important complementary work that fits these principles well.

### 4.1 Principle 1: CBK should be FAIR

We subscribe to the FAIR principles within the context of CBK,^2^ therefore CBK infrastructure should enable and promote FAIR knowledge. This is especially true of knowledge used for clinical decision support.

#### 4.1.1 Findable

Findable knowledge is categorized and tagged effectively such that others can discover it and relies on knowledge being available to all. Knowledge that is hard to find never competes in the marketplace of ideas. Broadly, technical design should support scalable virtual work platforms for collection, processing, analysis, visualization, and integration of knowledge object(s). Second, both technical and semantic interoperability must be considered earlier during the design for supporting the CBK knowledge repositories. The findability of knowledge objects should be supported by services that include unambiguous identifier schemes (such as Internationalized Resource Identifiers or IRIs) as well as the deployment of Application Programming Interfaces to increase the reuse of knowledge objects.

While gaps exist to make CBK findable, there is momentum to fill them. For example, there is no public “knowledge” registration system of record to catalog or index available CBK. Some funding agencies and publishers have realized the importance of the findability of the “digital assets” and making data stewardship to include long‐term care of such assets. For instance, the National Institutes of Health, has recognized the need and is seeking biomedical knowledgebase solutions that are, rightfully, separate from the existing data repositories.^10^

#### 4.1.2 Accessible

Open and accessible knowledge systems will manifest differently than data. Open knowledge systems will help people generate, access, understand, and reuse CBK with the opportunity to bring together community and stakeholders from diverse perspectives, yet there are still many unknowns. A lack of open knowledge or the lack of attention to open knowledge systems can hinder information dissemination, hamper equitable knowledge exchange, and delay decision‐making. Having open knowledge systems, however, does not offer quality assurances of the knowledge objects or knowledgebase and also can muddle intellectual property rights. As a result, accessibility, openness, and trustworthiness must be considered while building CBK infrastructure, not as an afterthought.

#### 4.1.3 Interoperable

Interoperability “…is the ability of different information systems, devices and applications (systems) to access, exchange, integrate and cooperatively use data in a coordinated manner, within and across organizational, regional and national boundaries, to provide timely and seamless portability of information and optimize the health of individuals and populations globally.”^11^ The Health Information Management Systems Society (HIMSS) definition focuses specifically on data. We posit that these concepts can be used to guide the development knowledge systems as well. Interoperability brings together the power of multiple systems and ideas but depends on conformance across multiple implementations. The presence of interoperable mechanisms can drive adoption of knowledge by reducing exchange barriers. Moreover, a modular, interoperable infrastructure architecture enables integration across systems for fostering the LHS.

Interoperability of knowledge will require explicit understanding across several dimensions, including values and value sets (ie, data), logic types, and knowledge expression types. Like data, these dimensions will require development and integration of standards of data and knowledge objects, which requires collaboration, coordination, incentives, and investments. Not having interoperable systems can impede progress through many means. Duplicating knowledge across systems, divergent knowledge, inaccurate representation of knowledge, delay in information availability, and loss of situational awareness or context can all prevent integration of computable knowledge. Yet, having interoperable systems still requires checks and cautions to reveal biases and assumptions and ensure data and knowledge are relevant and sufficient for decision‐making.

#### 4.1.4 Reusable

Reusability ensures that knowledge persists through time in a functional and meaningful way, and that knowledge models and ontologies evolve with the greater ecosystem. Reusability also addresses temporal concerns. Knowledge built on limited data may expand significantly as new technologies are created and matured or as new data and information become available. It is wise to ensure that systems dependent on CBK provide the user with the current state of that knowledge as well as its provenance. Depending on the techniques employed, the outputs of the machine learning models that may generate knowledge may lack reproducibility as to how they arrived at their result. This presents a problem for consuming validated knowledge prior to putting them in practice. In order to deploy in a clinical practice, fundamental activities should focus beyond creating new models and systems, and should include studying how to best repeat the meaning behind the output of knowledge generation. Yet, formidable hurdles exist to CBK reusability. They will need to address the lack of widely accepted and consumable standards as well as governance surrounding CBK.

### 4.2 Principle 2: Consumers need to know how trustworthy knowledge is

Trustworthiness at its core refers to something being truthful or honest as well as acting in accordance to those principles. Thus in the CBK context, trustworthiness^12^ applies to both what CBK is and what it does. A core question remains: How do I know that this CBK is correctly implemented and integrated, here and now, for this use, in this context? The issue is that the CBK itself is not entirely captured by metadata or provenance, involves both people and systems, and involves quality checks like self‐testing, monitoring, and logging. Thus, “trustworthiness” is complicated and provides a vast space for exploration. A CBK infrastructure that supports trustworthiness needs to be able to address questions like these:

**Knowledge:** Where did the knowledge come from? Is it what I think it is? Is it being used in the right way? Did it change and does it matter?

**Scale:** Can I use this CBK everywhere? Can I use it for different things? Can I use it over time, even as it changes?

**Ease of creation and evolution:** Also called $K\to K^{\prime }$. Can I implement appropriately trustworthy and scalable CBK, moving my model from bench to bedside? Can I share and let others extend my CBK?

**Sustainability:** Can I enforce ownership of the CBK I have created? Is my IP traceable as it evolves? Can I participate in communities that support its generation and distribution? Am I compliant with applicable law and regulation?

**Publishing/distribution/archiving:** How do I get others to find my CBK? Can I make it part of the scientific record? CBK is never static. It is created, distributed, regulated, modified, and applied in potentially novel scenarios. Moreover, an object or infrastructure may have a degree of trustworthiness that varies depending on its application. These bullet points highlight that “trustworthiness” is about more than the scientific validity and provenance of the core algorithm or implementation. There are of course more stakeholders than just creators (researchers) and consumers (patients). There are owners of the IP, publishers who need to sustain a distribution network, collaborators who change and extend CBK—all of them care about “trustworthiness.”

### 4.3 Principle 3: CBK and its infrastructure should be open

FAIR systems serve as the necessary core of CBK technical infrastructure, while openness ensures equity of access to that knowledge. Enabling open knowledge needs to build on open and transparent foundations.^6^ Much like data, we believe in having knowledge as open as possible and only closed as necessary. With government research funding agencies like the NIH requiring open release of both scientific findings (through open access publication policies) and data (through data sharing policies), the expectation of open knowledge has become a large part of the biomedical landscape. Computable knowledge should, in turn, also follow those mandates for exactly the same reasons more traditional knowledge is required to be open.

## 5 DISCUSSION

Through our discussions in the MCBK Technical Infrastructure Working Group (and now the Technical Standards and Infrastructure Working Group), three guiding principles emerged requiring consideration for developing a sustainable CBK infrastructure: Promote interoperable systems (a) within an accessible and open environment (b) that enable stable, trustworthy knowledge representations (c).

Knowledge is created, distributed, regulated, modified, and applied in potentially novel scenarios. When we think about what knowledge does, we can envision infrastructure that helps CBK to do things. Optimizing infrastructure could make it easier to integrate into existing health information systems—that helps CBK be used and reused in ways it was not originally designed and makes it easier to trust CBK at the point of use.

As noted in the sections above, knowledge in general—including CBK—constantly evolves, never remaining static. The interconnected relationship between data and knowledge (Data to Knowledge [D2K]) suggests the principles that apply to data are extensible for developing and maintaining knowledge objects. This continuum of D2K underscores the importance of system interoperability, which is essential for enhancing the value (eg, use and reuse) of the knowledge objects. Yet, as discussed, knowledge objects and the environment in which they evolve must accommodate the needs of knowledge objects to inform the LHS. Thus, we cannot assume the environment requirements for knowledge objects will be the same as with data objects.

Moreover, as the CBK definition implies, evidence can be subjective and not objective without a full‐spectrum, robust assessment of knowledge and gaps; “Infrastructure” should be value neutral, yet, the mere presence or absence of it affects value. How do we address this? And, who is going to pay for infrastructure? Does this approach make things easier or harder and for whom? Who benefits (or loses)? Does it both make the impossible possible and the possible easy? Does it drive adoption or slow it? Raise costs or lower them? These cavities of understanding pose more questions than answers.

It is important to note our distinction between open knowledge and open knowledge infrastructure in the principles. Knowledge infrastructure must provide a level playing field for access. However, there is some value in not excluding proprietary knowledge from such an environment. It will have to be up to the user of that knowledge to determine if the knowledge is trustable, even if it cannot be transparently evaluated. Furthermore, some forms of knowledge, like machine learning models, are opaque in their fundamental approach, but still have value.

A complete picture of building a CBK technical infrastructure comes with risks. If we do not make knowledge findable, we risk not having knowledge used or resources misspent through duplicative efforts. Yet, when we do make knowledge findable, the objects, attributes, and relationships may be applied incorrectly (ie, out of context) or overfitted (eg, creating associations where there is no causality). Ultimately, CBK technical infrastructure must support both the management of CBK in its various forms and the movement of CBK into practice, integration with existing systems, and appropriate and effective use.

## CONFLICT OF INTEREST

The authors declare no conflict of interest.

## Endnote

* Dr Bruce Bray is a Clinical Professor of Biomedical Informatics at the University of Utah, Salt Lake City, UT, USA.
